# In Pursuit of Theoretical Ground in Behavior Change Support Systems: Analysis of Peer-to-Peer Communication in a Health-Related Online Community

**DOI:** 10.2196/jmir.4671

**Published:** 2016-02-02

**Authors:** Sahiti Myneni, Nathan Cobb, Trevor Cohen

**Affiliations:** ^1^ School of Biomedical Informatics University of Texas Health Science Center at Houston Houston, TX United States; ^2^ Georgetown University School of Medicine Washington, DC United States

**Keywords:** behavior change, online social media, web interventions, smoking cessation

## Abstract

**Background:**

Research studies involving health-related online communities have focused on examining network structure to understand mechanisms underlying behavior change. Content analysis of the messages exchanged in these communities has been limited to the “social support” perspective. However, existing behavior change theories suggest that message content plays a prominent role reflecting several sociocognitive factors that affect an individual’s efforts to make a lifestyle change. An understanding of these factors is imperative to identify and harness the mechanisms of behavior change in the Health 2.0 era.

**Objective:**

The objective of this work is two-fold: (1) to harness digital communication data to capture essential meaning of communication and factors affecting a desired behavior change, and (2) to understand the applicability of existing behavior change theories to characterize peer-to-peer communication in online platforms.

**Methods:**

In this paper, we describe grounded theory–based qualitative analysis of digital communication in QuitNet, an online community promoting smoking cessation. A database of 16,492 de-identified public messages from 1456 users from March 1-April 30, 2007, was used in our study. We analyzed 795 messages using grounded theory techniques to ensure thematic saturation. This analysis enabled identification of key concepts contained in the messages exchanged by QuitNet members, allowing us to understand the sociobehavioral intricacies underlying an individual’s efforts to cease smoking in a group setting. We further ascertained the relevance of the identified themes to theoretical constructs in existing behavior change theories (eg, Health Belief Model) and theoretically linked techniques of behavior change taxonomy.

**Results:**

We identified 43 different concepts, which were then grouped under 12 themes based on analysis of 795 messages. Examples of concepts include “sleepiness,” “pledge,” “patch,” “spouse,” and “slip.” Examples of themes include “traditions,” “social support,” “obstacles,” “relapse,” and “cravings.” Results indicate that themes consisting of member-generated strategies such as “virtual bonfires” and “pledges” were related to the highest number of theoretical constructs from the existing behavior change theories. In addition, results indicate that the member-generated communication content supports sociocognitive constructs from more than one behavior change model, unlike the majority of the existing theory-driven interventions.

**Conclusions:**

With the onset of mobile phones and ubiquitous Internet connectivity, online social network data reflect the intricacies of human health behavior as experienced by health consumers in real time. This study offers methodological insights for qualitative investigations that examine the various kinds of behavioral constructs prevalent in the messages exchanged among users of online communities. Theoretically, this study establishes the manifestation of existing behavior change theories in QuitNet-like online health communities. Pragmatically, it sets the stage for real-time, data-driven sociobehavioral interventions promoting healthy lifestyle modifications by allowing us to understand the emergent user needs to sustain a desired behavior change.

## Introduction

Unhealthy behaviors such as smoking, physical inactivity, poor diet, and alcohol consumption contribute to 835,000 deaths in the United States annually [1,2] and are associated with an increased risk of chronic diseases such as hypertension, diabetes, stroke, and cancer [3]. Behavior modification is an important component of chronic disease management and sustainable healthy living. Adherence to healthy behaviors (eg, abstinence from smoking) requires a significant support infrastructure for long time intervals [4,5]. Research suggests that social relationships play an important role in an individual’s engagement in health issues [6-10]. While community-based social interventions harnessing the positive effects of social contacts exist [11-15], the mechanisms underlying the influence of social relationships on health behaviors are not fully understood. The ubiquity of online communities gives us invaluable datasets in the form of electronic traces of peer-to-peer communication, which may help us understand social influence and behavior. With the onset of mobility and connectivity in the communication sector, messages exchanged in health-related online communities reflect the intricacies of human health behavior as experienced in real time at individual, community, and societal levels.

Several studies on online social networks provide valuable insights into social influence, information spread, behavioral diffusion, and the structural aspects (who has ties to whom) [16-20]. Most prior research on social networks has made exclusive use of structure of social ties, where network structure is derived from the frequency of communication among members belonging to a network. Conversely, content analysis of online communities focused on specific behavior change mechanisms, social support [21-23], and emotional coping [24,25]. Prior qualitative studies on online community interactions have focused on (1) development and evaluation of network-based interventions [26,27], (2) user perceptions on utility of online communities for a specific health-related illness (eg, mental health [28]) and general conversational interests of specific population (eg, elderly [29,30]), (3) the role of online communities in identification of key quality indicators for patient-centered care [31], (4) effects of gamification features on overall technology acceptance [32], (5) users’ privacy concerns [33], (6) the quality of communication content in the online platforms [34,35], and (7) user participation patterns in network-based interventions [36-38]. Other qualitative studies examining communication content in online communities adopt a passive approach where researchers attempt to understand information-seeking patterns on websites or interactions in discussion groups [39]. Such studies have examined the help mechanisms and social support-related content of online self-help groups for alcoholism [40], cancer [41], and other health disorders such as Huntington’s disease [42]. Hwang et al conducted a network-based survey on the Sparkpeople forum, where members focus on a weight loss regimen [43]. The qualitative survey data were analyzed for social support themes using grounded theory techniques. Results indicated that the major social support themes were encouragement and motivation, information, and shared experiences [43]. The majority of research studies that have examined communication content in online communities have focused on assigning peer-to-peer communication events to various social support categories (eg, informational support, emotional support) [44]. However, social support is only one of numerous interpersonal mechanisms facilitated by the social ties established in online communities [45,46]. Existing theories of behavior change and patient engagement models suggest many different content-driven strategies to elicit specific sociobehavioral mechanisms beyond social support (eg, stimulus control, observational learning) to help users achieve a behavior change and self-manage an illness [47]. Different cognitive constructs in existing behavior change theories suggest different techniques [48]. Recent online survey research examined user perceptions of different social influence mechanisms to understand the relationship between network participation and smoking cessation self-efficacy [46]. Results of this survey have revealed that participation in health issue-specific social networking sites significantly influenced each social factor, which in turn resulted in greater smoking cessation self-efficacy. However, it is not known the extent to which any of the theoretically grounded strategies empirically manifest in the communication among network users [49]. Looking at the past and current trends of health-related online social networks, several research avenues can be pursued in order to strategize the use of the networks for improving health care. Advancing existing sociobehavioral theories, understanding fundamental mechanisms of behavior change, and formulating and evaluating novel interventional approaches are important avenues of research opened by these virtual platforms. Are theoretical models of sociobehavioral models of change applicable to both offline and online contexts? [49,50]. In-depth qualitative study of network interactions provides us with an unprecedented opportunity to refine existing theories and models of social networks, social support, and behavior-change that were formulated based on face-to-face communication.

In this paper, we describe the results of a grounded theory-based [51,52] content analysis of messages exchanged in an online social network for smoking cessation called QuitNet. We will use inductive coding techniques to (1) abstract and characterize the essence of peer-to-peer communication in online communities, and (2) understand how the identified themes relate to existing theoretical constructs and taxonomy of behavior change techniques. This approach will alter the current paradigm of studying behavior using online interactions from being hypothesis-driven [53] to being empirically grounded. Existing qualitative research conducted with a specific aspect of behavior change in mind is fundamentally different from our empirically driven, grounded theory approach. This analysis enhances our understanding of the manifestation of multiple behavior change constructs, which were formulated in the context of face-to-face communication using laboratory-based social science approaches, in the context of online social relationships.

### Theoretical Rationale

Several health behavior theories and models have been formulated to explain behavior change in general (see Table 1). These frameworks have served as guides for the development and evaluation of face-to-face and online interventions. Of the existing theoretical models, the Health Belief Model (HBM) [54,55], Theory of Reasoned Action (TRA) [56], the Transtheoretical Model (TTM) [57], and Social Cognitive Theory (SCT) [58] are found to be the most used in published smoking cessation intervention studies [59-62]. While each of these theoretical frameworks has its own merits and limitations, researchers have indicated concerns about the applicability of these models to consumers in the digital era [49,50]. The aforementioned four theories have been applied to the largest number of published studies on smoking cessation interventions.

#### The Transtheoretical Model of Change

TTM tries to explain the behavior change mechanisms by synthesizing several constructs drawn from other theories [57]. Stages and processes of change are the two main components of TTM. The former block explores the temporality of behavior change, while the latter encompasses cognitive and behavioral concepts such as decisional balance, self-efficacy, and rewards program. Precontemplation, contemplation, preparation, action, maintenance, and termination are the six stages of change, where each stage involves a process of progress.

#### The Theory of Reasoned Action

TRA suggests that the behavior of a person is determined by one’s behavioral intention [56]. Intent of a behavior is a function of the person’s attitude toward the behavior, their subjective norm associated with the behavior, and their perceived behavioral control.

#### Social Cognitive Theory

The SCT is a theory based on reciprocal determinism between a behavior, the environment, and a person [58]. This theory emphasizes self-efficacy, an important concept related to self-confidence. Self-efficacy is defined as “people’s judgments of their capabilities to organize and execute courses of action required to attain designated types of performances” p. 391 [58]. Current literature agrees on a common definition that self-efficacy “refers to what a person believes he or she can do on a particular task” p. 506 [58]. Goal attainment and confidence building through self-monitoring and continuous feedback is often used to improve a person’s self-efficacy. Other important constructs in SCT include behavioral capability, observational learning, reinforcement, outcome expectations and expectancies, emotional coping, and self-control. The construct of observational learning has been used by network scientists to provide an explanation for social influence and network clustering of people engaging in the same health behavior [58]. According to SCT, observational learning in behavior change occurs when an individual watches another person engage in a given behavior and receive reinforcements. Another component of SCT called reciprocal determinism takes into account the interactions among individuals, their environments, and behavior goals. The environment in SCT refers to a conglomeration of factors that are external to the individual including their social network (ie, family, friends, and peers) and physical objects that might affect behaviors. In the case of smoking, the physical objects can include availability of patches, access to smoking-designated areas in the work place, and so forth.

#### Health Belief Model

HBM is one of the most widely used conceptual frameworks for explaining and changing individual health behavior. HBM evolved from a cognitive theory perspective and is a value-expectancy theory, which attempts to explain and predict individual’s attitudes toward objects and actions [54]. Major components in HBM include perceived susceptibility, perceived severity, perceived benefits, perceived barriers, cues to action, and self-efficacy. An individual’s perceptions of a behavior can be used as predictors of behavior change outcomes under certain conditions that are dependent on demographic (eg, age, gender) sociopsychological (eg, personality, social class), and structural variables (eg, prior knowledge, experience).

#### Taxonomy of Behavior Change Techniques

Abraham et al defined a set of theory-linked behavior change techniques that can be used to characterize and differentiate between different types of intervention content [45]. Their taxonomy of 26 theory-linked techniques is the first step towards creating a model that provides a snapshot of intervention content in the context theory-driven behavior change constructs. A single behavior change technique can be related to similar behavior change processes from multiple theories. Consequently, the taxonomy integrates multiple behavioral theories [48] such as the theory of planned behavior [56], SCT [58], operant conditioning [63], and social support models on health-related behaviors [64]. As a result, the taxonomy provides a common vocabulary to understand the ways that sociobehavioral and cognitive constructs of the existing behavior change theories have been operationalized in a specific intervention.

**Table 1:** Theoretical constructs from behavior change theories (adapted from Revere & Dunbar [47]).

Theory	Concept	Definition
Health Belief Model	Perceived susceptibility	One’s opinion of chances of getting a condition
	Perceived severity	One’s opinion of how serious a condition and its consequences are
	Perceived benefits	One’s opinion of the efficacy of the advised action to reduce risk or seriousness of impact
	Perceived barriers	One’s opinion of the tangible and psychological costs of the action
	Cues to action	Strategies to activate readiness
	Self-efficacy	Confidence in ability to take action and persist in action
Stages of Change Model	Pre-contemplation	Unaware of problem, hasn’t thought about changes
	Contemplation	Thinking about changes
	Preparation	Making a plan to change
	Action	Implementations of a specific action plan
	Maintenance	Continuation of desirable actions, or repeating periodic recommended step(s)
	Consciousness raising	Increasing awareness via information, education, and personal feedback about the healthy behavior
	Dramatic relief	Feeling fear, anxiety, or worry because of the unhealthy behavior, or feeling inspiration and hope when they hear about how people are able to change to healthy behaviors
	Self-reevaluation	Realizing that the healthy behavior is an important part of who they are and want to be
	Environmental reevaluation	Realizing how unhealthy behavior affects others
	Social liberation	Realizing that society is more supportive of the healthy behavior
	Self-liberation	Believing in one’s ability to change and making commitments and recommitments
	Helping relationships	Finding people who are supportive of their change
	Counter-conditioning	Substituting healthy ways of acting and thinking for unhealthy ways
	Reinforcement management	Increasing the rewards that come from positive behavior and reducing those that come from negative behavior
	Stimulus control	Using reminders and cues that encourage healthy behavior as substitutes for those that encourage the unhealthy behavior
Theory of Planned Behavior and Theory of Reasoned Action	Behavioral intervention	Perceived likelihood of performing the behavior; prerequisites for action
	Attitude	One’s favorable or unfavorable evaluation of the behavior
	Behavioral belief	Belief that the behavioral performance is associated with certain attributes or outcomes
	Normative belief	Subjective belief regarding approval or disapproval of the behavior
	Subjective norm	Influence of perceived social pressure weighted by one’s motivation to comply with perceived expectations
	Perceived behavioral control	One’s perception of how easy or difficult it will be to act
Social Cognitive Theory	Reciprocal determinism	Behavior change results from interaction between individuals and environment
	Behavioral capability	Knowledge and skills to influence behavior
	Expectations	Beliefs about likely results of action
	Self-efficacy	Confidence in ability to take action and persist in action
	Observational learning	Beliefs based on observing others
	Reinforcement	Responses to a person’s behavior that increase or decrease chances of recurrence
	Emotional coping responses	Strategies or tactics that are used by a person to deal with emotional stimuli.

## Methods

### Materials

QuitNet is one of the first online social networks for health behavior change [65] and has been in continuous existence for the past 16 years. It is widely used with over 100,000 new registrants per year. QuitNet has members who are current and former smokers seeking to quit or stay abstinent. The members are globally distributed and come from over 160 countries including Canada, the United Kingdom, Australia, and South Africa. Previous studies on QuitNet indicated that participation in the online community was strongly correlated with abstinence [66]. The dataset studied in this paper was drawn from a previously studied quality improvement database and consists of de-identified messages in the public threaded forums, in which participants post messages and reply directly to each other. A database of 16,492 de-identified public messages from March 1-April 30, 2007, was used in our study. All messages were stripped of identifiers but re-coded for ego id (the individual posting) and alter id (the individual whose message is being replied to), self-reported abstinence status of sender and receiver, date, and position within the thread.

### Methods

The objective of this qualitative analysis was to characterize the nature of communication content exchanged by QuitNet members, thus capturing essential meaning of communication and factors affecting smoking cessation. This sort of analysis ultimately enables the abstraction of communication themes as they emerge from the data itself. Such inductive analysis is the principle technique used in the grounded theory method generating themes, where themes emerge from data itself [51,52]. Open coding and constant comparison are the two main characteristics of the analysis that can be used to ensure the derivation of meaningful representative themes from social network data. Open coding describes data by means of conceptual (rather than descriptive) codes, which are derived directly from the data, and constant comparison enables creation of precise and consistent codes by comparing these codes to observed phenomena and their contexts many times.

Often the messages exchanged among network members reflect a local language that is ingrained in the network’s unique culture. However, when it is interpreted out of context, they lose their context-specific meaning. Similarly, in a much more general sense, before the advent of Twitter (an online social networking and microblogging service), the word “tweeps” (defined as followers of a person/organization on Twitter) was never used. Interestingly, current trends suggest having a high number of “tweeps” as a metric to measure how well followed a person is. Emergence of local language is a commonly found feature of a community, and the same can be applied to online communities as well. Therefore, when analyzing online social network data to understand communication patterns underlying human behavior, understanding community-specific context is mandatory to derive meaningful inferences from the data.

A grounded theory approach was used to analyze QuitNet data to understand the core concepts, the interrelations among concepts, and the roles played by these concepts in an individual’s smoking cessation activity. The first step in the coding process involved open coding, where a line-by-line analysis was performed on the messages to derive abstract concepts from the data. The messages considered for analysis were selected at random using a scripted random number generator [67]. Each selected message was then reviewed, noting pertinent smoking cessation-related concepts in terms of general open codes that were generated dynamically as the data were reviewed.

Examples of open codes included “statistics,” “pregnancy,” “boredom,” “temper,” “patch,” and “pledge.” This process was repeated until no new concepts were produced from the dataset. Appropriateness of code assignment was ascertained using constant comparison, where instances of codes were compared in an iterative manner to make sure they reflected the same concept. The second step was performed by re-organizing and re-grouping the open codes using axial coding. Axial coding allowed for the identification of unifying, repeated patterns underlying the concepts and their relationships, thereby revealing core themes relevant to smoking cessation. Examples of core themes include “Family and friends,” “Obstacles,” and “Traditions.” Initial coding was performed manually, and later the NVivo software suite for qualitative analysis was used to analyze themes and their patterns of occurrence in the data. A total of 585 randomly selected messages were analyzed as described using grounded theory principles. Furthermore, the analysis was carried out for an additional 210 messages to ensure no new concepts emerged. Once themes were identified, a second coder conducted thematic analysis of a subset of 100 messages to ascertain the applicability of the derived themes to other QuitNet messages. This second round of coding was used to measure interrater reliability using Cohen’s kappa measure. This qualitative analysis allowed for an in-depth evaluation of the interactions among people in the QuitNet virtual community and thereby a deeper understanding of the behavior change processes that QuitNet users undergo when attempting to cease smoking.

This thematic taxonomy derived from our grounded theory analysis was then mapped to theoretical constructs derived from SCT, TTM, HBM, and TRA, since these theories had been applied to several published studies on smoking-related behavior change. In addition, we also used the original behavior change taxonomy, developed by Abraham et al, with 26 theory-linked behavior change strategies [45]. As part of theme-theory mapping, the messages in each theme and corresponding thematic definitions were compared to descriptions of theoretical constructs and taxonomy techniques in order to ascertain whether a particular theme facilitates the transmission of a specific behavior change construct.

## Results

###  QuitNet Themes

**Figure 1:**
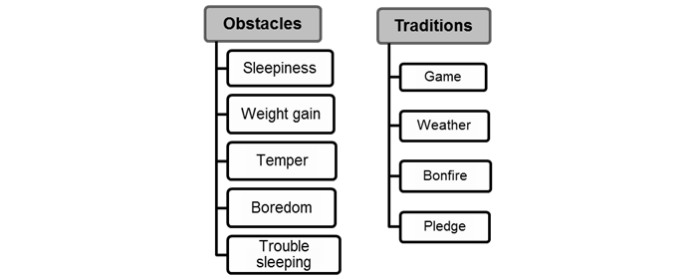
Themes in QuitNet.

A total of 43 different concepts were identified, which were then grouped under 12 themes. Examples of the grouping strategy employed to arrive at the thematic level are shown in Figure 1, where the “Obstacles” theme is composed by subsuming multiple concepts: “sleepiness,” “weight gain,” “temper,” “boredom,” and “trouble sleeping.” These concepts were cited as hurdles that members faced in their attempts to quit smoking. Similarly, “traditions,” “playing games,” “sharing weather details,” “attending virtual bonfire events,” and “taking part in daily online pledge” were the observed communal practices that are deeply rooted in the QuitNet community. Definitions of the themes and example messages for each theme are listed in Table 2.

**Table 2:** QuitNet themes, definitions, and example messages.

Theme	Definition	Example message
Quit Obstacles	Messages in which members talk about the hurdles they are dealing with or have dealt with to stay abstinent (eg, sleepiness, weight gain, temper)	I lost quits in the past because I was so mean and nasty that my family and friends told me to smoke.
Teachable Moments	Messages where the senders mention about the incentives one gets for not smoking in terms of quality of life	Food is wonderful...smell is wonderful...I smoked from 14-46...I never knew what I was missing.
Quit Readiness	Messages that attempt to provide inspiration and prompt readiness to quit and initiate a smoke-free life	You can do anything if you would want it bad enough...
Cravings	Messages that capture the real-time expressions of the users urge to smoke	I want a cigarette very much. I am trying to resist.
Conflict	Messages that reflect a rift between two group members	No one likes being called a liar, especially if they are NOT. Go sit
Relapse (confessions, reasons, retries)	Messages in which members explain why they relapsed and/or share their emotions after they suffered a relapse	I hate myself, I slipped again. I lighted the nicodemon
Traditions	Messages that focus exclusively on QuitNet-specific events such as bonfires, pledges, games, and so on	I’ve got over 5K unsmoked cigs which I’d be delighted to unload onto a raging bonfire.
Quit Progress	Messages in which members communicate their progress based on abstinence time and/or number of unsmoked cigarettes	Gratefully smoke free for 33 days, 17 hours, 1 minute and 6 seconds.
Family and Friends	Message in which members mention their spouses, children, or friends as motivators	My hubby...poor guy used to get to sleep when I smoked...now he is sleepless but smiling...
Virtual Rewards	Messages in which members mention the virtual gifts (such as bracelet, virtual pet, socks) received on QuitNet marking a milestone	awesome three days. I like the bracelet.
Social Support	Messages where the content reflects the elements of praise, advice, empathy, and guidance	Almost a year already.//// Congratulations to you, what a great accomplishment.
Pharmacotherapy	Messages where members explicitly discuss and evaluate various pharmacotherapy options and best practices for management of nitone withdrawal symptoms	I did not use any nrt though I recently went on welburtin after days ct

**Figure 2:**
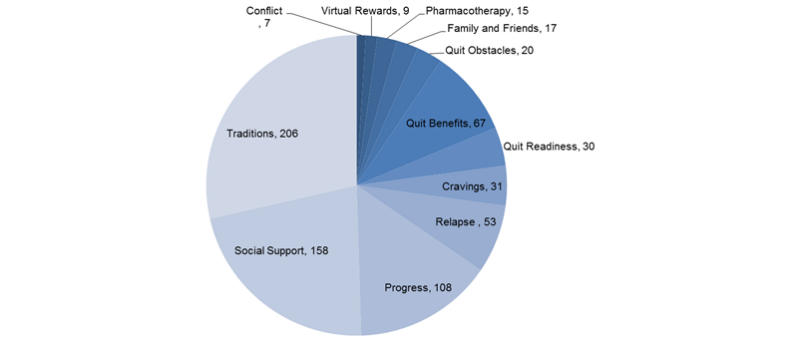
Grounded theory-based qualitative analysis of 795 messages.

A detailed distribution of the themes across messages is shown in Figure 2. “Traditions,” “Social support,” and “Progress” were the most frequently found themes, followed by discussions related to “Teachable moments/Benefits,” “Relapse,” and “Cravings,” “Conflict”-related messages were the least frequently found, only behind “Virtual Rewards,” “Pharmacotherapy,” “Family and friends,” and “Obstacles.”

QuitNet members exchange messages pertaining to traditions that are specific to QuitNet. Examples of traditions are as follows: (1) bonfire: a virtual event hosted regularly where members bring their unsmoked cigarettes and throw them into a fire, and (2) pledge: a member virtually extends their hand to another member indicating their commitment towards staying abstinent. This represents the support the member offers to the next person in line to help them stay smoke-free, and as such is one example of the content of messages belonging to the social support category. These messages provide guidance, express empathy, convey admiration, and promote bonding. Expressions of empathy, love, trust, and caring, which form the basis of emotional support, were also communicated using phrases such as “hugs,” “flowers,” and “kisses.” Members use measurable metrics such as the number of unsmoked days and cigarettes, the amount of money not spent on cigarettes, and the number of days of life saved by staying smoke-free to measure their “Progress.” These metrics are automatically calculated by the website using a user’s recorded quit date and displayed to the user and can be embedded in messages similar to an email signature. Members refer to these calculated metrics when providing positive feedback to others and utilize them for self-monitoring.

Analysis of the QuitNet data provided crucial insights into the relapse experiences of smokers and ex-smokers. Work-related stress, family tragedies, inability to ward off cravings, and a false notion of “just one puff” (denotes weak moments where members smoke a cigarette thinking that it would not affect their ability to stay abstinent from then on) were cited as common reasons for relapse. Relapse is a common problem encountered by smokers who are trying to quit and ex-smokers who successfully quit [68]. In addition to messages indicating risk factors for relapse, messages where members declare their relapse and communicate their emotions (eg, “tears rolling down,” “cheating the loved ones,” and “feeling like a loser”) after relapse were also included in this theme. Also, messages describing the “aha moments” where members recollect the reasons behind their decision to quit smoking occur in the dataset. Health-related issues such as the onset of smoking-related disease and pregnancy are cited as common drivers for these teachable moments/benefits, while quality of life concerns such as problems related to exercise, family time, physical appearance, and social awkwardness are also listed as reasons for quitting.

The day-to-day urges to smoke in QuitNet members’ journeys towards smoke-free lives were defined by cravings for cigarettes. This theme (“Cravings”) includes messages with content where successful quitters explained to fellow members how they dealt with cravings. Some messages even contained information about members’ experiences and efforts as they dealt with cravings in real-time. Messages relevant to the quit readiness theme displayed an effort made by QuitNet members to encourage fellow members by making inspiring, engaging, and thought-provoking comments on the role played by personality traits such as attitude and willpower in a successful quit attempt. Messages also have content through which members mentioned the obstacles they were facing, or have faced, at some point of their abstinence phase. Weight gain, temper, problems with sleep, and boredom were among these hurdles. Family (eg, spouse, children) and friends are mentioned in some of the messages as support network or motivators or obstacles. For instance, members mentioned not being able to stay abstinent because of watching their spouses smoke. Pharmacotherapy options are also discussed in QuitNet messages. Usage of patches and gums and going “cold turkey” (ie, quitting without any pharmaceutical assistance) are discussed as facilitators of behavior change. The members requested information about withdrawal effects and side-effects associated with the use of nicotine replacement therapies. Also, successful quitters advised newer members to make use of a patch to fight cravings and avoid relapse. Another emergent behavior exhibited by QuitNet members involved the role of virtual rewards. Some of these rewards included bracelets, virtual pets, socks, and access to an “elder lodge” where successful quitters meet virtually. Rewards were given when members met milestones such as 3-day, 15-day quit, and 100-day quits, 1-year anniversaries, and so forth.

### Interrater Reliability

Two researchers independently coded another subset of 100 randomly selected messages using the thematic terminology developed using grounded theory techniques. The codes they assigned to the messages had a Cohen’s kappa measure of 81.6%, where the 84 of the 100 messages had observed agreement. Disagreement was resolved using discussion, and the majority of the disagreement (12/16 discrepancies) was attributable to messages with “dual” content, where they could potentially be deemed as observing a community-specific tradition or measuring user progress in their smoking cessation efforts.

###  Thematic Interrelationships: Comparison With Existing Behavior Change Theories

The themes identified in QuitNet communication relate to the sociobehavioral and cognitive constructs of the existing behavior change theories. Tables 3 and 4 show how QuitNet themes can facilitate a driver of behavior change that relates to one of the theoretical constructs. A comparison matrix for inductively derived themes (seen in columns) and theoretically derived constructs (seen in rows) is provided based on the analysis of their definitions and the concepts they represent. An “X”-marked cell (see Tables 3 and 4) indicates that a given theme relates to a particular construct. For example, consider the concept of stimulus control, which involves using reminders and cues that encourage healthy behavior as substitutes for those that encourage the unhealthy behavior. For individuals who are accustomed to smoking early in the morning, there exists a QuitNet-specific tradition where members post messages describing early morning weather and reaffirm their commitment to stay abstinent. Themes such as virtual rewards, traditions, and progress have components that attempt to improve self-efficacy of an individual. An individual’s self-efficacy can affect their motivation to achieve a goal, such as adhering to a healthy behavior [17,18]. Persons with high self-efficacy are more likely to persist longer in efforts to achieve the desired goal [19,20]. In the case of smoking cessation, ability to ward off cravings and stay abstinent can be improved by enhancing a person’s self-efficacy [69-72], which can be achieved by setting and achieving short-term goals. Organizing such goals in a group environment also induces observational learning. For instance, virtual rewards such as bracelets and virtual pets accomplish the task of short-term goal setting. Watching other members receive these rewards often motivated QuitNet members to stay abstinent as evidenced by the following quote: “So proud of you, I won’t light my cigarette, want that lovely bracelet on my hand”. Bonfires (a component of the “Traditions” theme) are related to observational learning, where a QuitNet member is motivated by the praise another member received at the event on account of the number of unsmoked cigarettes they brought. Similarly, themes such as cravings and relapse address the aspect of dramatic relief described by the TTM. Teachable moments/benefits and obstacles relate to the decisional balance component of behavior change. Environmental reevaluation is also provided by these two themes. The social support theme includes several important constructs such as consciousness raising, cue to action, emotional coping, and helping relationships. For example, when a member was attempting to overcome a craving, other QuitNet members often post messages that attempt to help the peer member realize how far they have come, the reasons for their quitting, and provide them with a supporting shoulder.

**Figure 3:**
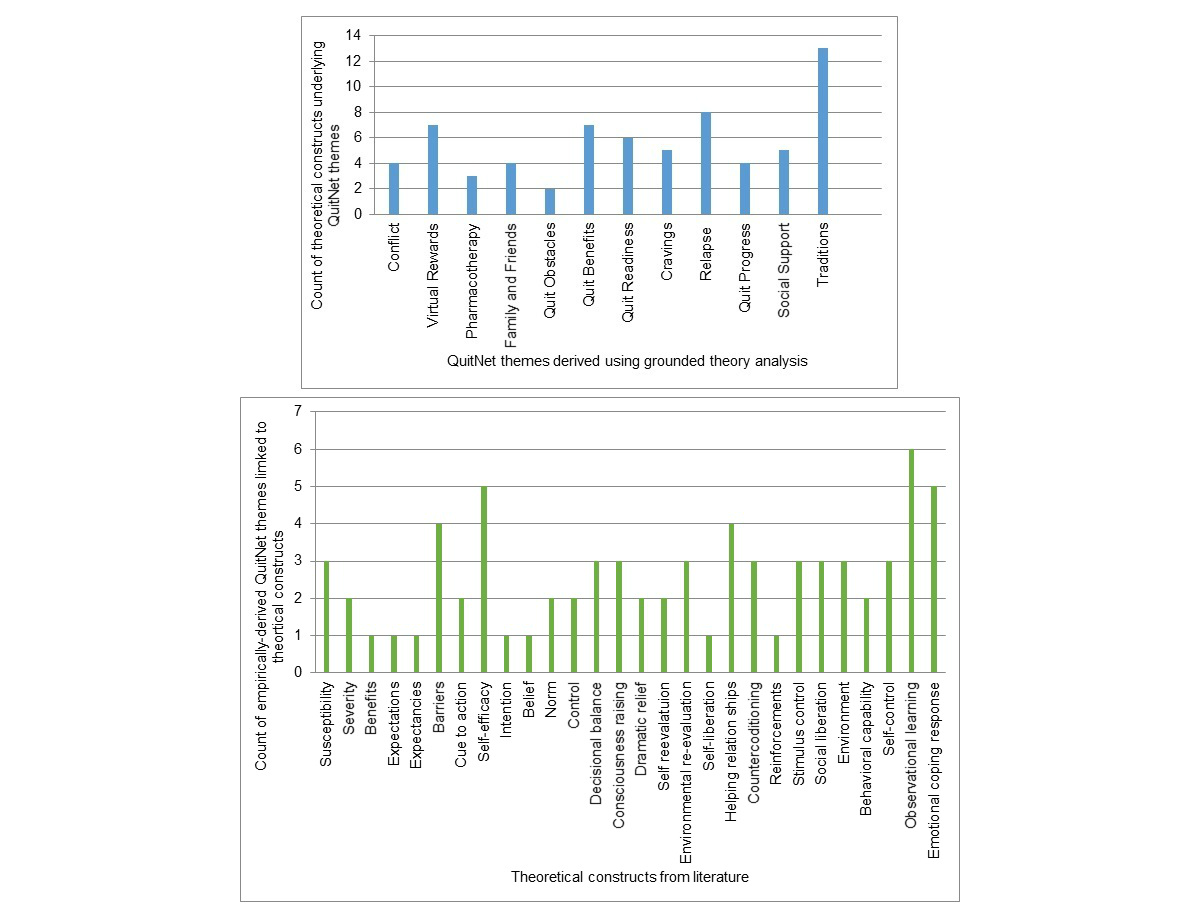
Thematic and theoretical prevalence in QuitNet content.

As described above, several constructs from the existing intra- and inter-individual behavior change theories are put together and compared with the themes derived from QuitNet content. The graphs in Figure 3 present the prevalence of the constructs in QuitNet themes. Self-efficacy and observational learning are the most relevant theoretical constructs, followed by observational learning and helping relationships (see Figure 3). On the other hand, traditions-related messages were highly aligned with theoretical constructs, followed by relapse, virtual rewards, and teachable moments/ benefits (Figure 3). Results indicate that community-based activities such as traditions organized in virtual communities such as QuitNet might play an important role in operationalizing theoretical constructs in the virtual settings. In addition, member-generated strategies such as bonfires and pledges facilitated the highest number of theoretical constructs from a variety of theories. Therefore, it is important to note that no single theory from behavioral science provides a basis for all of the themes emerging from QuitNet messages.

**Table 3:** Theme-theory matrix: conflict, virtual rewards, pharmacotherapy, family and friends, quit obstacles, and quit benefits.

Themes/Theoretical constructs	Conflict	Virtual rewards	Pharmacotherapy	Family and friends	Quit obstacles	Quit benefits
Susceptibility						X
Severity						X
Benefits						X
Expectations						
Expectancies						X
Barriers					X	
Cue to action			X			
Self-efficacy	X	X				
Intention						X
Belief						
Norm	X					
Control						
Decisional balance					X	X
Consciousness raising	X		X			
Dramatic relief						
Self-reevaluation						X
Environmental re-evaluation	X			X		X
Self-liberation						
Helping relationships	X			X		
Counterconditioning		X	X			
Reinforcements		X				
Stimulus control		X				
Social liberation	X			X		
Environment		X		X		
Behavioral capability						
Self-control		X				
Observational learning		X		X		
Emotional coping response				X		

**Table 4:** Theme-theory matrix: quit readiness, cravings, relapse, quit progress, social support, and traditions.

Themes/Theoretical constructs	Quit readiness	Cravings	Relapse	Quit progress	Social support	Traditions
Susceptibility	X		X			
Severity	X					
Benefits						
Expectations			X			
Expectancies						
Barriers		X	X		X	
Cue to action					X	
Self-efficacy	X		X	X		X
Intention						
Belief	X					
Norm						X
Control	X					X
Decisional balance		X				
Consciousness raising					X	
Dramatic relief		X	X			
Self-reevaluation						X
Environmental re-evaluation						
Self-liberation						X
Helping relationships			X		X	X
Counterconditioning						X
Reinforcements						
Stimulus control				X		X
Social liberation	X					
Environment						X
Behavioral capability			X			X
Self-control				X		X
Observational learning		X	X	X		X
Emotional coping response		X	X		X	X

### Mapping to Taxonomy of Behavior Change Techniques

**Figure 4:**
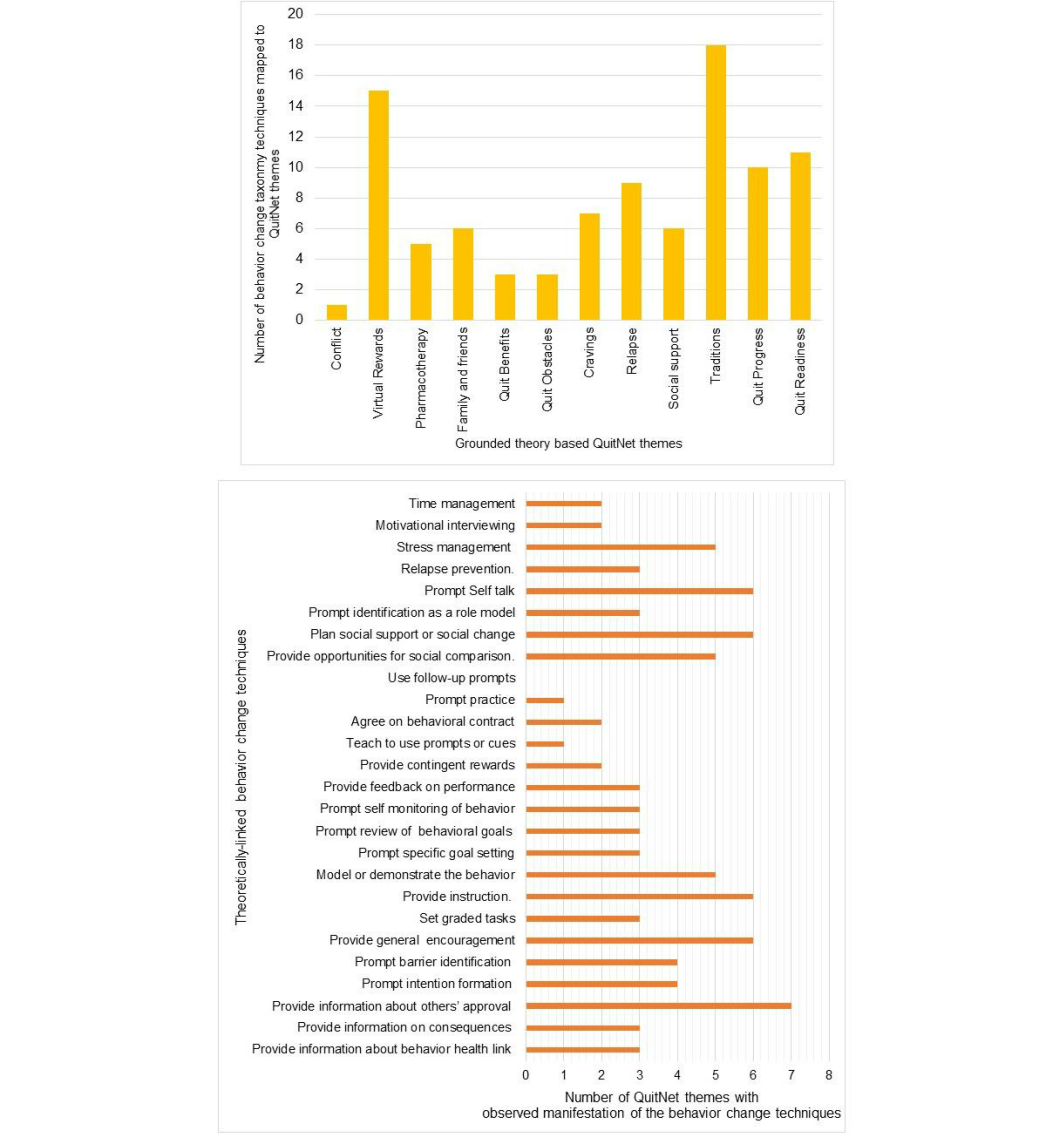
Taxonomy-based analysis of QuitNet themes.

The themes identified in QuitNet communication relate to the 21 standardized theory-linked behavior change techniques put together by Abraham et al. Tables 5-7 show the manifestation of the behavior change techniques in QuitNet themes. A binary matrix for inductively derived themes (seen in columns) and theoretically linked constructs (seen in rows) is provided based on the comparative analysis of their definitions and the concepts they represent. A tick mark (see Tables 5-7) indicates that a given theme operationalized a specific behavior change technique. For example, quit obstacles, cravings, and relapse are the three themes that embedded content to prompt barrier identification among QuitNet users. Similarly, providing general information about the risks associated with smoking cessation (eg, mortality-related information) has been facilitated through exchange of messages that belong to quit obstacles, quit benefits, and quit readiness. As shown in Figure 4, almost all (25/26, 96%) the behavior change techniques are found to be operationalized through messages exchanged by QuitNet users. Follow-up prompting, which is implicitly embedded in peer communication in QuitNet, was found to be the only technique where a specific QuitNet theme cannot be matched. Of the remaining 25 techniques in the taxonomy, we found that proving social approval, social support, prompting self-talk, providing instructions, and general encouragement were most commonly facilitated in QuitNet communication. Conversely, messages categorized under traditions were transmitting content that can operationalize many (18/26, 69%) behavior change techniques. Interestingly, from our analysis on theme-theory-comparison, we found that traditions-related messages were aligned with the highest number of theoretical constructs. All QuitNet themes derived using grounded theory techniques were found to be facilitating at least one behavior change technique.

**Table 5:** Theme-taxonomy matrix: conflict, virtual rewards, pharmacotherapy, and family and friends.

Behavior change techniques	Conflict	Virtual rewards	Pharmaco-therapy	Family and friends
Provide information about behavior health link	–	–	–	–
Provide information on consequences	–	–	–	–
Provide information about others’ approval	–	✓	–	✓
Prompt intention formation	–	–	–	✓
Prompt barrier identification	–	–	–	–
Provide general encouragement	–	✓	–	✓
Set graded tasks	–	✓	–	–
Provide instruction	–	–	✓	–
Model or demonstrate the behavior	–	✓	–	✓
Prompt specific goal setting	–	✓	–	–
Prompt review of behavioral goals	–	✓	–	–
Prompt self monitoring of behavior	–	–	✓	–
Provide feedback on performance	–	–	–	–
Provide contingent rewards	–	✓	–	–
Teach to use prompts or cues	–	–	✓	–
Agree on behavioral contract	–	✓	–	–
Prompt practice	–	–	–	–
Use follow-up prompts	–	–	–	–
Provide opportunities for social comparison	–	✓	–	✓
Plan social support or social change	–	✓	–	✓
Prompt identification as a role model	–	✓	–	–
Prompt self-talk	✓	✓	–	–
Relapse prevention	–	–	✓	–
Stress management	–	–	✓	–
Motivational interviewing	–	–	–	–
Time management	–	–	–	–

**Table 6:** Theme-taxonomy matrix: quit obstacles, quit benefits, cravings, and relapse.

Behavior change techniques	Quit obstacles	Quit benefits	Cravings	Relapse
Provide information about behavior health link	✓	✓	–	–
Provide information on consequences	✓	✓	–	–
Provide information about others’ approval	–	–	✓	✓
Prompt intention formation	–	✓	–	–
Prompt barrier identification	✓	–	✓	✓
Provide general encouragement	–	–	–	–
Set graded tasks	–	–	–	–
Provide instruction	–	–	✓	✓
Model or demonstrate the behavior	–	–	✓	–
Prompt specific goal setting	–	–	–	–
Prompt review of behavioral goals	–	–	–	–
Prompt self monitoring of behavior	–	–	–	–
Provide feedback on performance	–	–	–	✓
Provide contingent rewards	–	–	–	–
Teach to use prompts or cues	–	–	–	–
Agree on behavioral contract	–	–	–	–
Prompt practice	–	–	–	–
Use follow-up prompts	–	–	–	–
Provide opportunities for social comparison	–	–	–	✓
Plan social support or social change	–	–	✓	✓
Prompt identification as a role model	–	–	–	–
Prompt self-talk	–	–	–	✓
Relapse prevention	–	–	✓	✓
Stress management	–	–	✓	✓
Motivational interviewing	–	–	–	–
Time management	–	–	–	–

**Table 7:** Theme-taxonomy matrix: quit progress, social support, traditions, and quit readiness.

Behavior change techniques	Quit progress	Social support	Traditions	Quit readiness
Provide information about behavior health link	–	–	–	✓
Provide information on consequences	–	–	–	✓
Provide information about others’ approval	–	✓	✓	✓
Prompt intention formation	–	–	✓	✓
Prompt barrier identification	–	–	–	✓
Provide general encouragement	✓	✓	✓	✓
Set graded tasks	✓	–	✓	–
Provide instruction	–	✓	✓	✓
Model or demonstrate the behavior	✓	–	✓	–
Prompt specific goal setting	✓	–	✓	–
Prompt review of behavioral goals	✓	–	✓	–
Prompt self monitoring of behavior	✓	–	✓	–
Provide feedback on performance	✓	–	✓	–
Provide contingent rewards	–	–	✓	–
Teach to use prompts or cues	–	–	–	–
Agree on behavioral contract	–	–	✓	–
Prompt practice	–	–	✓	–
Use follow-up prompts	–	–	–	–
Provide opportunities for social comparison	✓	–	✓	–
Plan social support or social change	–	✓	✓	–
Prompt identification as a role model	✓	–	✓	–
Prompt self-talk	✓	–	✓	✓
Relapse prevention	–	–	–	–
Stress management	–	✓	–	✓
Motivational interviewing	–	✓	–	✓
Time management	–	–	✓	✓

## Discussion

### Study Implications

In the case of QuitNet, activities such as pledges and bonfires emerged from within the community and each of those events marks a specific aspect of the smoking-cessation process. With the evolution of communication channels from being traditional face-to-face conversations to virtual social networks powered by Web-based mHealth systems, the validity of existing behavior change theories in the digital era has been questioned [49,50]. This qualitative analysis establishes the validity of behavior change theories in the context of 21^st^-century technologies. In addition, the inductive evaluation of social network content revealed new sociocognitive constructs, which have not been considered by existing behavior change theories. For instance, the conflict theme, which deals with the conflicts that arise between QuitNet members, is not found in any existing sociobehavioral theories because the foundation for these theories is group cooperation and not group competition. This finding highlights the need to incorporate mechanisms that build trust among members who communicate with one another using virtual channels. In addition to emphasizing progress and positive aspects of smoking cessation, focus on community-building and social togetherness (eg, bonfires) have helped members adhere to their quit attempts. As another example, none of the messages mentioned the role of a physician in their efforts to cease smoking, suggesting the behavior change effort in this community is primarily self-propelled. Like any other virtual community, most content embeds aspects of social support. In addition to support, several other sociobehavioral elements related to behavior change theories were found in QuitNet messages. Our analysis revealed that most QuitNet themes (1) relate to important behavior change constructs belonging to multiple theories and (2) operationalize several techniques outlined in the behavior change taxonomy.

Qualitative methods form a very important toolkit to conduct nuanced analysis of health-related communications in online platforms. As we have shown, inductive analytic techniques that are data-driven enabled us to characterize peer interactions in QuitNet. Further, use of grounded theory analysis allowed us to develop thematic representations of QuitNet messages that are empirically driven and not theoretically biased. Subsequently, comparison analysis consisting of (1) sociobehavioral constructs from existing behavior change theories and (2) theoretically linked taxonomy of behavior change techniques allowed us to understand the theoretical roots and operational features of consumer-driven QuitNet communication. The methodological process itself is immensely informative, comprehensive, and generalizable, while being empirically grounded and theoretically aligned simultaneously. The applicability of the methods discussed in this paper can be taken well beyond the analysis of a small scale sample through use of automated text analysis methods. Communication exchanges in online communities are time-stamped and digitized and therefore are amenable to machine learning [73-75]. Classification of conversational and informational postings on social media websites has been attempted using a combination of human coding, statistical analysis, and machine learning [76]. Methods of distributional semantics have also been combined with machine learning algorithms to classify consumer health webpages based on language use patterns [77]. Semantic space models, methods of distributional semantics where both terms and larger units of text are represented in a high-dimensional vector space [78], have been applied to peer-to-peer interactions in online communities [79-83]. While much of this work is at an early stage, the adaptation of such methods of distributional semantics to represent communication between members of online communities shows considerable promise. As part of our ongoing and future studies, we have adapted methods of distributional semantics in conjunction with machine learning algorithms to enable high-throughput analysis of online social network data [82,83]. Such methods facilitating resource-optimized extension of qualitative analysis to large-scale digital health data can extend the research and application frontiers of social media, thereby further enhancing their positive impact on health-related behaviors.

###  Limitations

This paper provides insights into the ways that consumer interactions in online communities can be conducted using methods that are empirically motivated and theoretically driven. Such qualitative analysis provided useful insights into prominent themes in QuitNet communication. Although we have ensured the study of coding and reliability was conducted before we formulated theme-theory linking as a potential next step in order to minimize the influence of predispositional knowledge on theme identification, the analysis may be amenable to subjective knowledge. In addition, manual coding is highly labor-intensive and time-consuming. Consequently, the analysis is limited to a small sample size, potentially limiting the generalizability of these results. It is possible that given the low fraction of messages thematically coded, the distribution of the themes might not have been accurately represented. To attempt to address this, 210 messages were coded to reach thematic saturation. However, it may be possible that the remainder of the dataset contains additional themes that were not captured. The rapid growth of digital technologies will further complicate this issue, as it will generate a data deluge of millions of messages transmitted over the Web and mobile media. Therefore, for large datasets, one needs to complement the qualitative method with an automated technique that can optimize resource utilization. The QuitNet dataset considered in our analysis was recorded in 2007. For future studies, we will attempt to obtain further data drawn from recent datasets. However, we strongly believe that the findings from the reported data on human behavior still hold, since the basic tenet of forum-based communication (structure and logistics) remains the same. Even emerging health-related network platforms (eg, PatientsLikeMe) also embed online forums to facilitate peer-to-peer communication. Threaded discussions in the form of comments in contemporary platforms also provide forum-like environments to facilitate text-based communication among users in online platforms. In addition, this analysis does not take into account seasonal patterns that might affect an individual’s behavior change (eg, New Year’s resolutions) because of the limited size and time period of our QuitNet dataset. We will attempt to address these issues through use of larger longitudinal datasets. Similarly, there have been some novel developments with respect to modes of nicotine intake (eg, e-cigarettes) [84] that were not observed in our dataset. However, we do not believe the mechanisms of social influence in a forum-based context have changed substantially over the past decade. Proliferation of daily use social networks like Facebook and Twitter engage users in peer communication through modes other than text-based messaging. These open domain social networks facilitate study of online behavior through “likes” and “shares,” which may have provided additional insights [85,86] but were not a feature of our dataset. Consequently, our analysis is limited to text-based user communication among users of an online community.

###  Conclusions

This paper describes a qualitative analysis of online social network communication using a grounded theory approach. The key contributions of this study are as follows:

1. The study describes the first grounded theory–based qualitative analysis of the communication in an online social network developed to promote behavior change. Contrary to prior qualitative studies that focused on a specific behavior change mechanism (either social support or emotional coping), our paper presents an empirically driven perspective on manifestations of theoretical behavior change constructs in online platforms.
2. The methodological process allows investigation of consumer-driven behavior change attempts in online communities from the perspective of existing behavior change theories.
3. The study attempts to understand the applicability of hypothesis-driven behavior change constructs to organically evolving user communication in online platforms.
4. This is the first reported attempt of analyzing online user interactions using the taxonomy of behavior change techniques, subsequently imposing the structure of common vocabulary to understand the ways that consumer interactions implicitly operationalize sociobehavioral and cognitive constructs of existing behavior change theories.

Capturing the essence of the meaning underlying the messages exchanged during different situations and contexts in this manner provides important information to guide further investigations. Qualitative analysis of communication between members of an online social network can provide valuable insights into the mechanisms underlying human behavior change. With the onset of mobile phones and ubiquitous Internet connectivity, online social network data reflect the intricacies of human health behavior as experienced by real people in real time. Therefore, analysis of these data can also provide us with the much needed theoretical and empirical foundations for the design of effective intervention strategies. This study offers insights into the various kinds of behavioral constructs prevalent in the messages exchanged among QuitNet users. In addition, it underlines the need for the use of inductive approaches for the analysis of online social network data to capture community-specific culture. As such, these findings suggest the need for an aggregation of multiple theoretical constructs from more than one inter- and intra-individual theory. Given the context-rich nature of the messages, they yield empirical understanding of human behavior change. This understanding has important implications for both theory and practice. Theoretically, inductive analysis of virtual communities provides us with a basic understanding of human behavior in the digital era. In terms of practical implications, the study sets the stage for (1) modeling supervised machine learning algorithms that can scale the theoretically valid findings to large datasets [82] and (2) simulating social network models where the relations between members were inductively derived [82,83]. The study thus facilitates the development of data-driven digital health interventions that promote healthy lifestyle modifications by harnessing social influence mechanisms. Such transdisciplinary, theoretically validated, empirically grounded solutions may hold the key to a healthier future.

## Abbreviations

HBM: Health Belief Model
TRA: Theory of Reasoned Action
TTM: Transtheoretical Model
SCT: Social Cognitive Theory
